# Regional left atrial conduction velocity in the anterior wall is associated with clinical recurrence of atrial fibrillation after catheter ablation: efficacy in combination with the ipsilateral low voltage area

**DOI:** 10.1186/s12872-022-02881-6

**Published:** 2022-11-01

**Authors:** Shiou Ohguchi, Yasuya Inden, Satoshi Yanagisawa, Rin Fujita, Kenichiro Yasuda, Ken Katagiri, Mitsutoshi Oguri, Toyoaki Murohara

**Affiliations:** 1 Department of Cardiology, Kasugai Municipal Hospital, Kasugai, Japan; 2 Department of Cardiology, Nagoya University Graduate School of Medicine, Nagoya, Japan; 3 Department of Advanced Cardiovascular Therapeutics, Nagoya University Graduate School of Medicine, 65 Tsurumai-cho, Showa-ku, 466-8550 Nagoya, Aichi Japan

**Keywords:** LA conduction velocity, Atrial fibrillation, Catheter ablation, Low voltage area, Recurrence

## Abstract

**Background:**

Left atrial (LA) conduction velocity (CV) is an electrical remodeling parameter of atrial fibrillation (AF) substrate. However, the pathophysiological substrate of LA-CV and its impact on outcomes after catheter ablation for AF have not been well evaluated.

**Methods:**

We retrospectively evaluated 119 patients with AF who underwent catheter ablation and electroanatomical mapping during sinus rhythm. To measure regional LA-CV, we took triplet sites (A, B, and C) on the activation map and calculated the magnitude of the matched orthogonal projection vector between vector-AB and vector-AC, indicating two-dimensional CV. The median of the LA-CVs from four triad sites in both the anterior and posterior walls was set as the ‘model LA-CV’. We evaluated the impact of the model LA-CV on recurrence after ablation and relationship between the model LA-CV and LA-low voltage area (LVA) of < 0.5 mV.

**Results:**

During the 12-month follow-up, 29 patients experienced recurrence. The LA-CV model was significantly correlated with ipsilateral LVA. The lower anterior model LA-CV was significantly associated with recurrence, with the cut-off value of 0.80 m/s having a sensitivity of 72% and specificity of 67%. Multivariable analysis revealed that the anterior model LA-CV (hazard ratio, 0.09; 95% confidence interval, 0.01–0.94; p = 0.043) and anterior LA-LVA (hazard ratio, 1.06; 95% confidence interval, 1.00–1.11; p = 0.033) were independently associated with AF recurrence. The anterior LA-LVA was mildly correlated with the anterior model LA-CV (r = -0.358; p < 0.001), and patients with both lower LA-CV and greater anterior LA-LVA based on each cut-off value had the worst prognosis. However, decreased LA-CV was more likely to be affected by the distribution pattern of the LVA rather than the total size of the LVA.

**Conclusion:**

Decreased anterior LA-CV was a significant predictor of AF recurrence and was a useful electrical parameter in addition to LA-LVA for estimating AF arrhythmogenicity.

**Supplementary Information:**

The online version contains supplementary material available at 10.1186/s12872-022-02881-6.

## Introduction

Catheter ablation is an effective method for treating atrial fibrillation (AF) with the maintenance of sinus rhythm. Pulmonary vein isolation (PVI) is an essential approach, and its electric isolation from the left atrium (LA) achieves a high rate of freedom from AF. However, AF recurrence often occurs, which is not only due to pulmonary vein (PV) reconnection but also an additional arrhythmogenic atrium responsible for AF maintenance [1, 2]. Atrial substrate remodeling progresses with aging, the persistence of AF, heart failure, and cardiovascular risk factors [3–5]. The effect of remodeling is associated with intra-atrial electrical injury. Although the atrial low voltage area (LVA), defined by the bipolar amplitude, is an electrical remodeling index and a clinical tool that can be used to grasp AF condition during catheter ablation [6–9], there is still debate as to whether the LVA can be associated with AF vulnerability and the arrhythmogenic substrate to be targeted. In contrast, intra-atrial conduction velocity (CV) is another electrical parameter used to estimate the extent of atrial electrical remodeling and AF arrhythmogenicity [10]. However, little is known about the impact of atrial CV on outcomes.

In the present study, we evaluated regional CV using a mathematical method to calculate the matched orthogonal LA-CV vector between two vectors from the triad of the LA site on electroanatomical mapping. This study aimed to evaluate how the model LA-CV impacts AF recurrence after catheter ablation and how LA-CV represents AF substrate linked to the LA-LVA.

## Materials and methods

### Study population

The present study retrospectively analyzed a catheter ablation database at Kasugai Municipal Hospital in Japan. A total of 119 consecutive patients with symptomatic refractory paroxysmal AF (n = 57) and persistent AF (n = 62) who underwent radiofrequency catheter ablation between December 2016 and April 2019 were included. Indications for catheter ablation for AF and definition of paroxysmal and persistent AF were based on recent guidelines [11, 12]. The exclusion criteria for this study were as follows: (i) severe valvular heart disease; (ii) previous ablation treatment; (iii) follow-up period < three months after ablation; (iv) non-radiofrequency, cryoballoon ablation treatment; (v) failure to restore sinus rhythm by cardioversion after PVI; (vi) insufficient number of mapping points (< 1,000 points) in the LA; and (vii) impossibility to discontinue antiarrhythmic drug administration after a blanking period of three months following the procedure. This study was approved by our institutional ethics committee. All patients provided written informed consent prior to the procedure. All examinations and procedures were performed in compliance with the principles of the Declaration of Helsinki.

### Catheter ablation and post-procedural follow-up

To prepare for catheter ablation, each three-dimensional LA anatomical structure was constructed using 64-sliced computed tomography (CT) imaging. Transthoracic and transesophageal echocardiography were performed to assess cardiac information, including the presence or absence of LA thrombus before the procedure. Antiarrhythmic drugs were discontinued for at least five half-lives before catheter ablation.

All ablation procedures including PVI were performed under the guidance of the 3D-electroanatomical mapping system (CARTO, Biosense Webster) with an LA geometry obtained from the CT image [13]. Intravenous heparin (100 IU/kg) was administered immediately after the puncture and was added properly to maintain an activated clotting time of 300–350 s during the procedure [14, 15]. We used a 3.5 mm tip, open-irrigated contact force sensing ablation catheter (25–35 W, 42 °C, irrigation flow rate of 17–30 mL/min; Navistar ThermoCool SmartTouch, Biosense Webster, Inc.), a circular mapping catheter for PVI (Lasso, Biosense Webster, Inc., USA), and a multi-electrode mapping catheter for 3D-electroanatomical mapping (PentaRay, Biosense Webster, Inc., USA) [13]. The contact force of the catheter was targeted at 10–25 g, and catheter stability was set with a force-time integral of > 100 g*s. PVI was confirmed by the elimination of PV potentials recorded by a circular mapping catheter and bi-directional electrical block between the LA and PV [13]. When the AF rhythm persisted after PVI, external cardioversion was performed to restore the sinus rhythm. After the procedure, atrial arrhythmia was induced by rapid atrial stimulus pacing with a high dose of isoproterenol infusion, followed by focal ablation for extra PV origin and linear ablation for the provoked AF or atrial flutter (AFL) were added, if necessary.

All patients were followed up through the outpatient clinic for one year after discharge and were checked for AF recurrence. They were instructed to see their attending physician at one to two weeks and one month after discharge, followed by every three months for one year. At each follow-up consultation, they underwent 12-lead surface electrocardiography and were asked if they had any symptoms related to the presence of arrhythmia. Moreover, 24 h Holter monitoring was also performed at least one, three, and 12 months after discharge. AF recurrence was defined as an episode of AF or atrial tachycardia lasting ≥ 30 s on examination testing after a blanking period of three months following the procedure.

### Electrophysiological mapping

Mapping was performed using a multi-electrode mapping catheter (Pentaray, Biosense Webster, Inc.). Approximately 1,000–2,500 points per patient were obtained on LA during sinus rhythm after PVI and before the induction of atrial arrhythmia to avoid the effect of isoproterenol. Cardioversion was performed to restore sinus rhythm in the case of AF sustaining after PVI, and a 15 min interval was taken before the mapping to avoid any untoward effect by the cardioversion [16]. LA was divided into anterior and posterior sites, which were segmented by three borderlines; the two lines orthogonal to the mitral valve from the bottom of the left- and right-inferior PVs, and one line between the roofs of the left- and right-superior PVs. The definition of LVA was the area of electrogram amplitude (< 0.5 mV) in the bipolar voltage mapping, which was further divided into moderate LVA (mLVA: area of amplitude of 0.1–0.5 mV) and severe LVA (sLVA: area of amplitude < 0.1 mV). In addition, the presence of LVA was defined as areas covering > 5.0 cm^2^ of the LA in which LVA formation was classified as patchy, concentric, or combined (Supplemental Fig. 1).

### Analysis of LA-CV

**Fig. 1:**
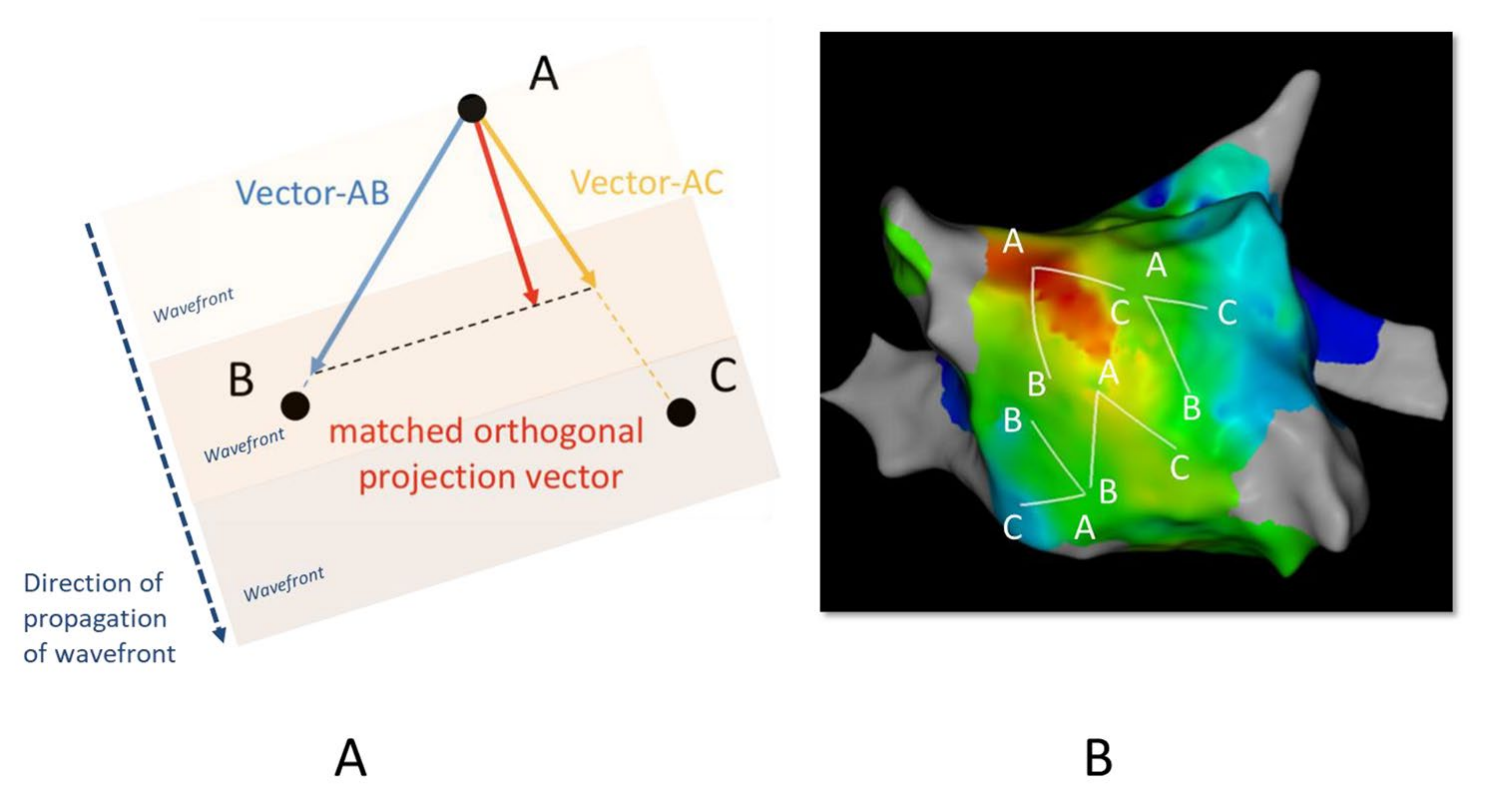
Analysis of LA-CV. (A) Local triplet points (A, B, and C) were set to make a triad region for measurement of the LA-CV vector along the direction of wave front propagation with avoidance of visual collision in the activation map. When point A was defined as the earliest point in this triangle, vector-AB and vector-AC were calculated for one-dimensional CV with the activation time and distance between points A and B or C. Then, we incorporated cartesian and trigonometric equations to calculate the magnitude of matched components between the vector-AB and vector-AC as forming the orthogonal projection, which resulted in velocity specifically in the two-dimensional direction of activation. (B) Four matched projection vectors were analyzed at different triad regions of each anterior and posterior LA wall. The medians of the LA-CVs from the four triad sites were set as ‘model LA-CVs’ in both anterior and posterior walls. These regions for analysis must be randomly selected and evenly distributed in the whole anterior and posterior regions and not to be inclined toward one side, respectively. CV, conduction velocity; LA, left atrium

We measured the magnitude of regional LA-CV by calculating the CV vector in the electroanatomical mapping obtained during sinus rhythms after PVI. The median of the magnitudes of the vectors was defined as the ‘model LA-CV’, which was from different LA triangle locations along the conduction route. This analysis was performed offline. The investigator was blinded to the outcome and the LVA map information of the patient. The bipolar atrial electrogram of the coronary sinus with a maximum slope was set as the time reference to take the local activation time at each obtained point [17]. The color-coded 3D activation sequence map was acquired using this method, in which the red color was identified as the earliest activation area, and the purple color was identified as the latest. Local triplet points (A, B, and C) were set to create a triad region for the measurement of the LA-CV vector along the direction of wave front propagation while avoiding visual collision in the activation map. When point A was defined as the earliest point in this triangle, vector-AB and vector-AC were calculated for one-dimensional CV with activation time and distance between points A and B or C, and for the degree of an angle between them with three distances among points A, B, and C measured with the module of the 3D-electroanatomical mapping system and cosine formula mathematically. Then, we incorporated trigonometric equations to calculate the magnitude of the matched component between the vector-AB and vector-AC to form the orthogonal projection, which resulted in the velocity of the two-dimensional direction of activation (Fig. 1 A) [17]. Because the surface of the atrial endocardium is not flat and the triplet region should be selected based on a two-dimensional presumed vector with flat space, we set each inter-site distance to be three points < 30 mm to minimize the dimensional error as much as possible. Overall, each point A was chosen not to overlap around the upper, lateral, lower, and septal area in front of and behind the LA, while points B and C were determined obliquely from point A to maintain the balance of the distance to share the entire LA body in total points. Four matched projection vectors were analyzed at different triad regions of each anterior and posterior LA wall. The medians of the LA-CVs from the four triad sites were set as the ‘model LA-CVs’ in both anterior and posterior walls. These regions for analysis must be randomly selected and evenly distributed throughout the anterior and posterior walls and not inclined toward one particular side (Fig. 1B).

### Statistical analysis

Statistical analysis was performed using JMP 5.1 Statistics. The clinical characteristics that were continuous variables are presented as the mean ± standard deviation. Categorical variables are presented as numbers (percentages). The *χ*^*2*^ test or Fisher’s exact test was used to compare categorical variables. Student’s *t*-test for normally distributed data or the Mann-Whitney *U* test for non-normally distributed data were used when differences were determined. The receiver operating characteristic (ROC) curves were plotted to assess the model LA-CVs and absolute value of the LA-LVA in the anterior and posterior regions for AF recurrence after ablation, and the areas under the curves (AUC) were compared. The cut-off values of AF recurrence in these parameters were determined based on the ROC curve. Kaplan-Meier survival curve analysis was used to estimate the prognosis, and the difference in the curves was assessed using a log-rank test. Pearson’s correlation coefficient analysis was used to evaluate the relationship between the model LA-CV and absolute value of the LA-LVA. The prognostic value of each factor for AF recurrence was evaluated using univariate Cox regression analysis. Significant factors in the univariate analysis were entered into a multivariate Cox regression model to identify the independent predictors. Statistical significance was set at a p-value of < 0.05.

## Results

### Baseline data and clinical characteristics with and without recurrence of AF

In the total population, 29 patients (nine patients with paroxysmal AF and 20 with persistent AF) experienced recurrence during the one year follow-up after ablation. Among these patients, 25 had AF recurrence, 1 had cavotricuspid isthmus AFL, 2 had peri-mitral AFL, and 1 had both peri-mitral and roof-dependent AFL. The baseline characteristics of patients with and without recurrence are shown in Table 1. There was a significant difference in persistent AF, LA dimension, and LA appendage (LAA) emptying velocity between the two groups. Patients with recurrence had lower anterior model LA-CV (0.74 ± 0.23 m/s vs. 0.89 ± 0.24 m/s, p = 0.005), greater anterior LA-LVA (18.7 ± 13.0 cm^2^ vs. 9.1 ± 5.9 cm^2^, p = 0.001), and posterior LA-LVA (9.7 ± 7.0 cm^2^ vs. 5.0 ± 4.5 cm^2^, p = 0.002) than those without recurrence. As far as the differences in the model LA-CV and LA-LVA between the anterior and posterior walls are concerned, both parameters in the anterior wall were significantly lower and greater than those in the posterior wall (0.85 ± 0.25 m/s vs. 0.95 ± 0.25 m/s, p = 0.020, and 11.5 ± 9.1 cm^2^ vs. 6.2 ± 5.6 cm^2^, p < 0.001, respectively).

**Table 1:** Baseline characteristics in the clinical recurrence and non-recurrence groups

Parameters	No recurrence N = 90	Clinical recurrence N = 29	P-value
Age (years)	68.0 ± 8.9	69.0 ± 7.0	0.523
Gender (male/female, %)	57/33 (63.3)	18/11 (62.1)	0.902
Body mass index (kg/m^2^)	23.5 ± 5.1	22.9 ± 5.5	0.569
Current or former smoker (%)	49 (54.4)	14 (48.3)	0.563
Persistent AF (%)	42 (46.7)	20 (69.0)	0.037
Comorbidity			
Type 2 diabetes mellitus (%)	14 (15.6)	6 (20.7)	0.571
Hypertension (%)	48 (53.3)	18 (62.1)	0.410
Dyslipidemia (%)	33 (36.7)	6 (20.7)	0.111
Chronic kidney disease (%)	33 (36.7)	10 (34.5)	0.831
Coronary artery disease (%)	7 (7.8)	3 (10.3)	0.704
Congestive heart failure (%)	25 (27.8)	7 (25.0)	0.773
Previous stroke (%)	13 (14.4)	6 (20.7)	0.400
CHADS2 score	1.5 ± 1.2	1.7 ± 1.0	0.564
CHA2DS2-VASc score	2.6 ± 1.6	3.0 ± 1.3	0.258
Medication at admission (%)			
ACE inhibitor or ARB (%)	44 (48.9)	14 (48.3)	0.954
Beta-blocker (%)	48 (53.3)	14 (48.3)	0.635
Echocardiography			
LA dimension (mm)	40.0 ± 5.8	42.7 ± 7.5	0.049
LV ejection fraction (%)	64.0 ± 13.6	67.4 ± 11.9	0.228
E/e’	12.6 ± 5.0	11.9 ± 3.2	0.375
LAA emptying velocity (m/s)	48.6 ± 20.9	39.0 ± 20.0	0.049
Primary measurements			
Anterior LA-LVA (cm2)	9.1 ± 5.9	18.7 ± 13.0	0.001
Posterior LA-LVA (cm2)	5.0 ± 4.5	9.7 ± 7.0	0.002
Anterior model LA-CV (m/s)	0.89 ± 0.24	0.74 ± 0.23	0.005
Posterior model LA-CV (m/s)	0.95 ± 0.24	0.92 ± 0.29	0.575
Coefficient of variation in anterior LA-CV (m/s)	0.46 ± 0.44	0.39 ± 0.42	0.458
Coefficient of variation in posterior LA-CV (m/s)	0.57 ± 0.63	0.46 ± 0.42	0.400

Values are expressed as mean ± standard deviation or number (percentage). AF, atrial fibrillation; ACE, angiotensin converting enzyme; ARB, angiotensin II receptor blocker; LA, left atrium; LV, left ventricular; LAA, left atrial appendage; LVA, low voltage area; CV, conduction velocity

A comparison of the ablation procedure between the recurrence and non-recurrence groups according to the type of AF is shown in Table 2. PVI was successfully achieved in all patients, and there were no significant differences in any procedure between the groups.

**Table 2:** Procedural parameters in the clinical recurrence and non-recurrence groups

Ablation procedures	No clinical recurrence N = 90	Clinical recurrence N = 29	P-value
Paroxysmal AF	N = 48	N = 9	
Pulmonary vein isolation (%)	48 (100)	9 (100)	N/A
LA leaner ablation (%)	0 (0)	1 (11.1)	0.158
Mitral isthmus ablation (%)	0 (0)	1 (11.1)	0.158
SVC isolation (%)	1 (2.1)	0 (0)	0.842
Non-PV foci ablation (%)	10 (20.8)	2 (10.0)	0.614
Cavotricuspid isthmus ablation (%)	48 (100)	9 (100)	N/A
Other additional ablation (%)	1 (2.0)	0 (0)	0.842
Persistent AF	N = 42	N = 20	
Pulmonary vein isolation (%)	42 (100)	20 (100)	N/A
LA leaner ablation (%)	6 (14.3)	5 (25.0)	0.245
Mitral isthmus ablation (%)	0 (0)	2 (10.0)	0.100
SVC isolation (%)	0 (0.0)	1 (5.0)	0.323
Non-PV foci ablation (%)	11 (26.2)	3 (15.0)	0.259
Cavotricuspid isthmus ablation (%)	42 (100)	20 (100)	N/A
Other additional ablation (%)	0 (0)	1 (5.0)	0.323
Procedure time (min)	210.3 ± 47.0	224.5 ± 42.0	0.152

Values are expressed as number (percentage). AF, atrial fibrillation; LA, left atrium; PV, pulmonary vein; SVC, superior vena cava

### Assessment of the model LA-CV and its impact on the outcome after ablation according to the cut-off value

**Fig. 2:**
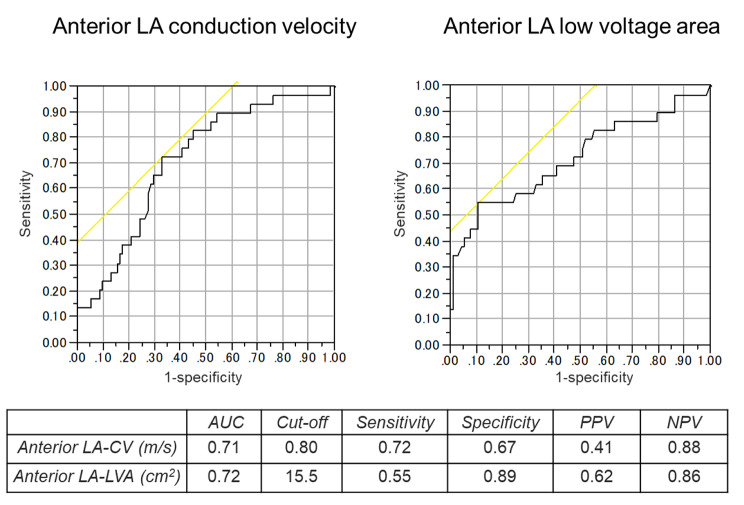
ROC curves and cut-off points of LA-CV (A) and LA voltage area (B) for associations with AF recurrence after catheter ablation. AF atrial fibrillation; AUC, area under the curve; CV, conduction velocity; LA left atrium; NPV, negative predictive value; PPV, positive predictive value; ROC, receiver operating characteristic

**Fig. 3:**
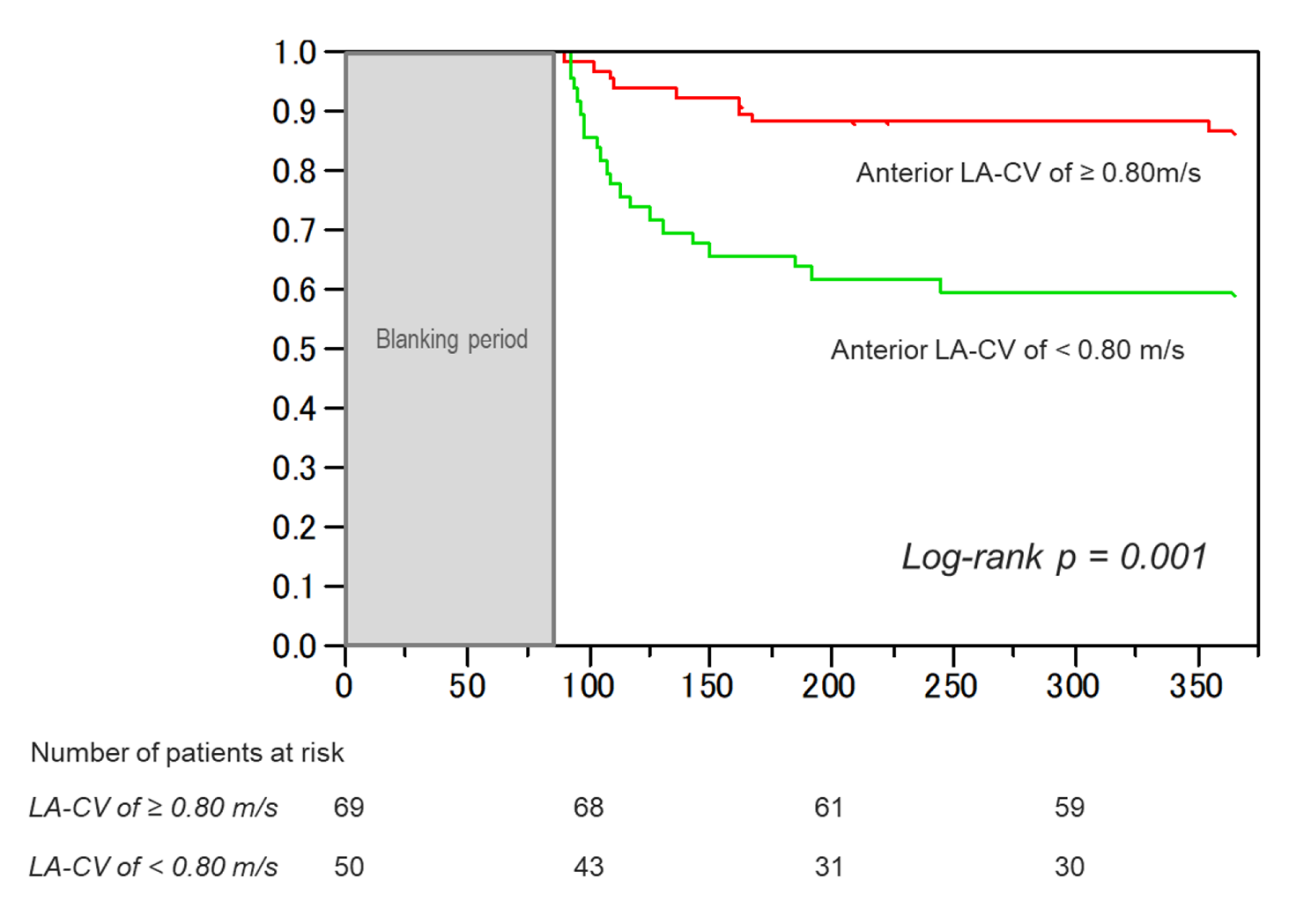
Kaplan-Meier curves of the survival-free rate of AF recurrence after ablation according to the cut-off point of the anterior model LA-CV of 0.80 m/s. AF, atrial fibrillation; LA-CV, left atrial conduction velocity

ROC curve analysis determined the cut-off value of the anterior model LA-CV for recurrence after catheter ablation as 0.80 m/s (AUC 0.71, sensitivity 72%, specificity 67%, positive predictive value [PPV] 41%, negative predictive value [NPV] 88%) (Fig. 2). A comparison of the baseline characteristics between patients divided according to the cut-off value of the anterior model LA-CV of 0.80 m/s are shown in Table 3. The patients with LA-CV < 0.80 m/s (n = 50) had a higher prevalence of persistent AF, CHA2DS2-VASc scores, and greater anterior LA-LVA than those with LA-CV ≥ 0.80 m/s (n = 69). Kaplan-Meier survival curve analysis demonstrated a significantly lower rate of AF event-free survival after ablation in patients with anterior model LA-CV < 0.80 m/s than in those with anterior model LA-CV ≥ 0.80 m/s (p = 0.001) (Fig. 3). The patterns of AF recurrence in patients with anterior model LA-CV < 0.80 m/s consisted of 18 patients with AF recurrence, 1 with cavotricuspid isthmus AFL, and 1 with both peri-mitral and roof-dependent AFL.

**Table 3:** Comparison of baseline characteristics between the patients with anterior model LA-CV ≥ 0.80 m/s and those with LA-CV < 0.80 m/s

Parameters	Anterior model LA-CV ≥ 0.80 m/s N = 69	Anterior model LA-CV < 0.80 m/s N = 50	P-value
Age (years)	67.6 ± 9.4	69.0 ± 7.1	0.370
Gender (male/female, %)	48/21 (69.6)	27/23 (54.0)	0.083
Body mass index (kg/m^2^)	23.8 ± 4.8	22.8 ± 5.7	0.299
Current or former smoker (%)	30 (55.6)	15 (53.6)	0.864
Persistent AF (%)	27 (39.1)	35 (70.0)	0.001
Comorbidity			
Type 2 diabetes mellitus (%)	8 (11.6)	12 (24.0)	0.074
Hypertension (%)	40 (58.0)	26 (52.0)	0.518
Dyslipidemia (%)	21 (30.4)	18 (36.0)	0.523
Chronic kidney disease (%)	26 (37.7)	17 (34.0)	0.680
Coronary artery disease (%)	4 (5.8)	6 (12.0)	0.318
Congestive heart failure (%)	17 (24.6)	15 (30.6)	0.472
Previous stroke (%)	8 (11.6)	11 (22.0)	0.126
CHADS2 score	1.4 ± 1.0	1.8 ± 1.3	0.161
CHA2DS2-VASc score	2.5 ± 1.5	3.1 ± 1.6	0.036
Medication at admission (%)			
ACE inhibitor or ARB (%)	32 (46.4)	26 (52.0)	0.545
Beta-blocker (%)	38 (55.1)	24 (48.0)	0.446
Echocardiography			
LA dimension (mm)	40.3 ± 6.5	41.2 ± 6.1	0.419
LV ejection fraction (%)	65.1 ± 13.3	64.5 ± 13.2	0.788
E/e’	12.1 ± 4.8	13.0 ± 4.4	0.324
LAA emptying velocity (m/s)	49.1 ± 21.1	42.5 ± 20.4	0.109
Primary measurements			
Anterior LA-LVA (cm2)	9.2 ± 7.3	14.5 ± 10.5	0.003
Posterior LA-LVA (cm2)	5.4 ± 4.3	7.2 ± 6.8	0.093

Values are expressed as mean ± standard deviation or number (percentage). AF, atrial fibrillation; ACE, angiotensin converting enzyme; ARB, angiotensin II receptor blocker; LA, left atrium; LV, left ventricular; LAA, left atrial appendage; LVA, low voltage area; CV, conduction velocity

### Relationship of the LA-LVA with clinical outcomes and the model LA-CV

ROC curve analysis was also performed for AF recurrence in terms of the LA-LVA, and the cut-off value of anterior LA-LVA was 15.5 cm^2^ (AUC 0.72, sensitivity 55%, specificity 89%, PPV 62%, NPV 86%) (Fig. 2). The anterior LA-LVA was mildly correlated with the anterior model LA-CV (r = -0.358; p < 0.001), while the posterior LA-LVA was weakly correlated with the posterior model LA-CV (r = -0.239; p = 0.009).

### Predictive value for recurrence after catheter ablation

Univariate Cox regression analysis identified that the persistent AF, anterior model LA-CV, anterior LA-LVA, posterior LA-LVA, LAA emptying velocity, and LA dimension were significant parameters for recurrence following ablation. On multivariate analysis, the anterior model LA-CV (hazard ratio [HR], 0.09; 95% confidence interval [CI], 0.01–0.94; p = 0.043) and anterior LA-LVA (HR, 1.06; 95% CI, 1.00-1.11; p = 0.033) remained independent factors of AF recurrence (Table 4).

**Table 4:** Predictors of clinical recurrence after ablation in the total population in univariate and multivariate cox regression analyses

Parameters	Univariate analysis		Multivariate analysis	
	HR (95% CI)	P-value	HR (95% CI)	P-value
Persistent AF	1.51 (1.03–2.29)	0.032		
Anterior model LA-CV	0.05 (0.01–0.35)	0.002	0.09 (0.01–0.94)	0.043
Posterior model LA-CV	0.71 (0.13–3.15)	0.661		
Anterior LA-LVA	1.08 (1.05–1.11)	< 0.001	1.06 (1.00–1.11)	0.033
Posterior LA-LVA	1.09 (1.04–1.14)	0.001		
LAA emptying velocity	0.98 (0.95–1.00)	0.034		
LA dimension	1.06 (1.00–1.13)	0.044		

AF, atrial fibrillation; LA, left atrial; CI, confidence interval; CV, conduction velocity; HR, hazard ratio; LVA, low voltage area; LAA, left atrial appendage

### Impact on the outcome after catheter ablation according to the cut-off value combined with the anterior model LA-CV and ipsilateral LVA

**Fig. 4:**
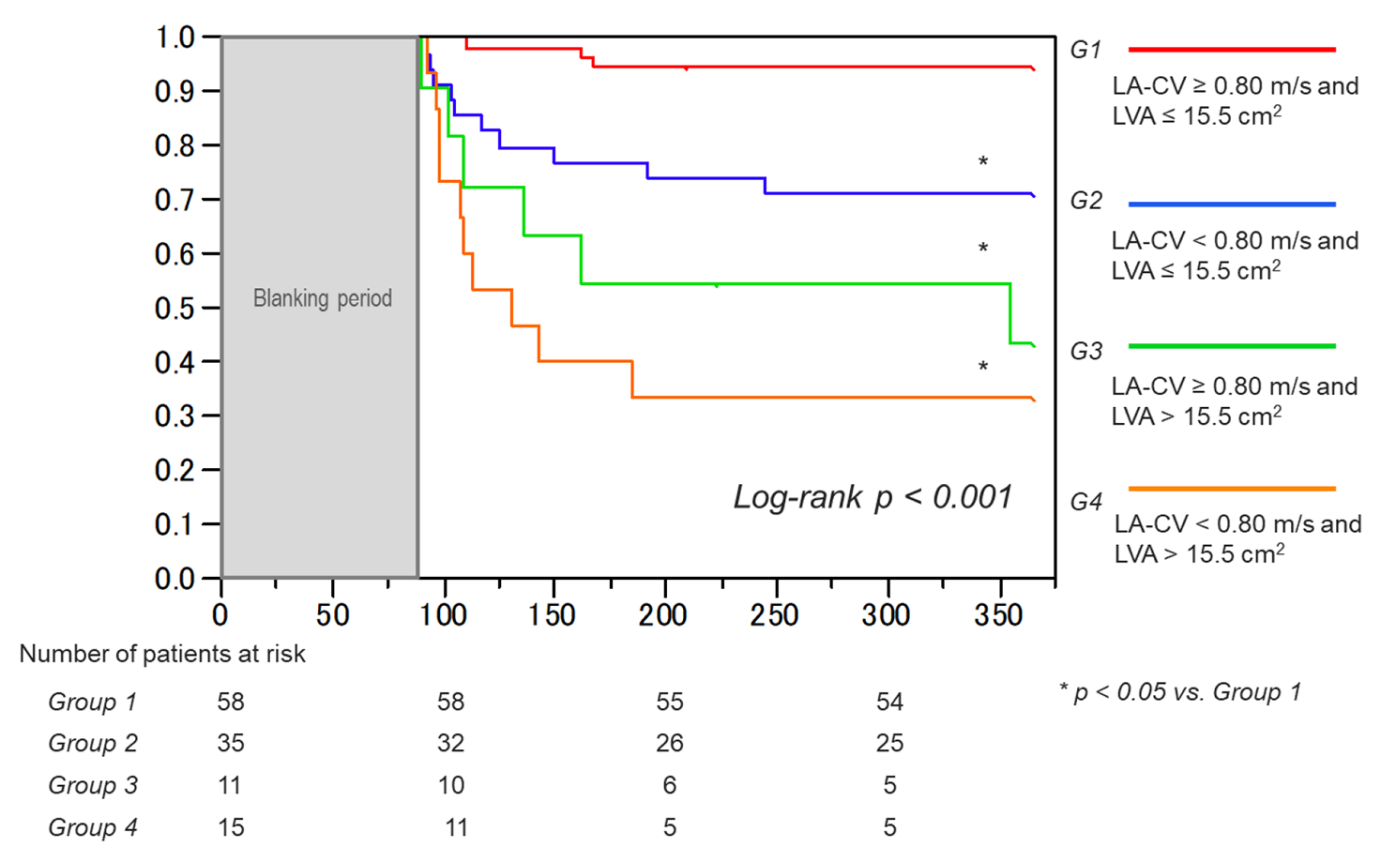
Kaplan-Meier curves of the survival-free rate from AF recurrence in the four groups according to two cut-off points (anterior model LA-CV value of 0.80 m/s and anterior LA-LVA value of 15.5 cm2). AF, atrial fibrillation; LA-CV, left atrial conduction velocity; LA-LVA, left atrial low voltage area

We classified the study subjects into four groups according to the above two significant cut-off values as follows: group 1 (anterior model LA-CV ≥ 0.80 m/s and anterior LA-LVA ≤ 15.5 cm^2^; n = 58), group 2 (anterior model LA-CV < 0.80 m/s and anterior LA-LVA ≤ 15.5 cm^2^; n = 35), group 3 (anterior model LA-CV ≥ 0.80 m/s and anterior LA-LVA > 15.5 cm^2^; n = 11); and group 4 (anterior model LA-CV < 0.80 m/s and anterior LA-LVA > 15.5 cm^2^; n = 15). Prognosis after AF ablation was compared among these groups. Kaplan-Meier survival curve analysis demonstrated a significant difference in the AF event-free survival rate after ablation among the four groups (p < 0.001), and group 4 showed the lowest survival rate compared to the other groups (Fig. 4). Furthermore, group 2 with decreased LA-CV showed a significantly lower survival rate than group 1 with normal LA-CV, despite the smaller LVA of ≤ 15.5 cm^2^ observed in both groups.

The anterior LA areas classified by the degree of the mean voltage amplitude (sLVA, mLVA, and non-LVA) were demonstrated in each group (Supplemental Fig. 2 A). The mean size of the sLVA was not very different between groups 1 and 2 and groups 3 and 4, which maintained a similar condition in the LA-LVA. The sizes of the mLVA and non-LVA were also not significantly different between groups 1 and 2, but the size of the mLVA was relatively larger in group 4 than in group 3. The distribution patterns of the anterior LA-LVA in the groups with an LVA > 5.0 cm^2^, that is, patchy, concentric, and combined, were observed with the groups with an LVA ≤ 5.0 cm^2^ (Supplemental Fig. 2B). Patchy and concentric patterns were dominant in groups 1 and 2, but patchy patterns were more frequently found in group 1, whereas concentric patterns were greater in group 2. In contrast, the combined pattern was the major form of the LA-LVA in groups 3 and 4. Patchy and concentric patterns were rarely observed in groups 3 and 4.

### Outcomes of the anterior model LA-CV in patients with paroxysmal and persistent AF

**Fig. 5:**
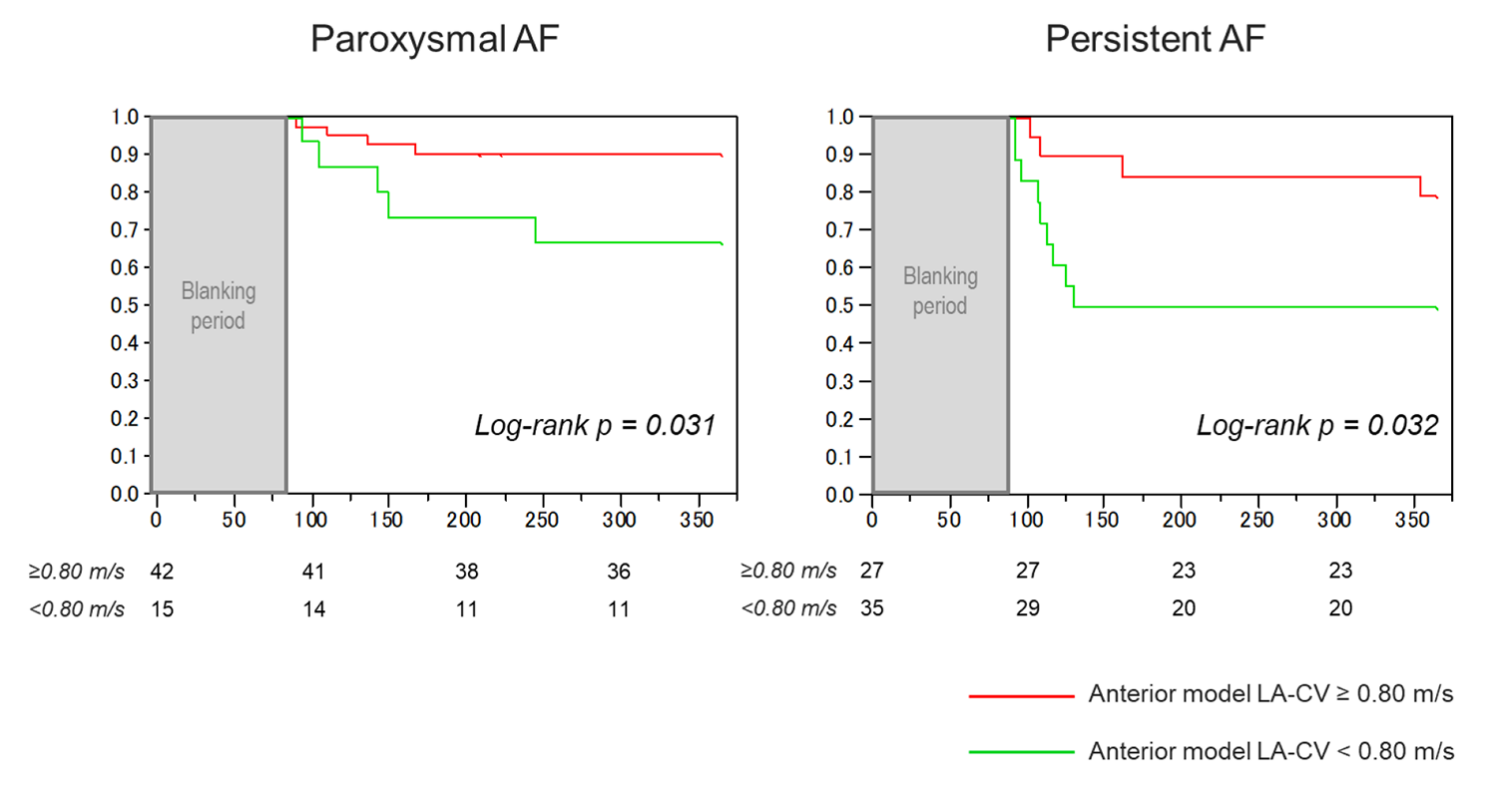
Kaplan-Meier curves of the survival-free rate of AF recurrence after ablation according to the cut-off point of the anterior model LA-CV of 0.80 m/s in patients with paroxysmal AF and persistent AF. AF, atrial fibrillation; LA-CV, left atrial conduction velocity

We evaluated the prognostic value of the decreased anterior model LA-CV of < 0.80 m/s in the population divided by the type of AF. There were significant differences between patients with paroxysmal AF and those with persistent AF in the anterior LA-CV (0.93 ± 0.27 m/s vs. 0.78 ± 0.20 m/s, p = 0.001), and LA-LVA (9.6 ± 7.2 cm^2^ vs. 13.2 ± 10.4 cm^2^, p = 0.030). Fifteen patients (26%) with paroxysmal AF and 27 patients (44%) with persistent AF had a reduced anterior model LA-CV of < 0.80 m/s. In both the paroxysmal and persistent AF groups, patients with an anterior model LA-CV of < 0.80 m/s demonstrated a significantly worse prognosis for recurrence after ablation than in those with an anterior model LA-CV of ≥ 0.80 m/s (Fig. 5).

## Discussion

The association between LA-CV and AF has not been well studied, while heterogeneity in regional CVs is speculated to be a critical substrate for functional reentry at AF onset. However, the criteria for CV measurement are unclear and not standardized. Kurata et al. calculated anterior and posterior LA-CVs with distance and conduction time from the start to the end of the propagation wave front in the LA activation map and revealed an association between anterior LA-CV and AF recurrence after catheter ablation [18]. Sato et al. recently reported that slower global LA-CV on an imaging system was associated with AF recurrence after PVI [19]. Although other CV measurement methods include the value calculated between geodesic linear pole pairs or in triad areas with minimum interelectrode distance along the wave front path [13, 20–22], they are considered to indicate local or regional CV, which only show limited electrical characteristics in LA. Our unique model of the magnitude of the matched orthogonal projection vector calculated in the triangle area can indicate regional CV and can also represent a gravel CV by evaluating the median of LA-CVs among four different sample sites in both the anterior and posterior walls separately. The analysis of the method is simple and does not require complex interpretation but has a theoretical basis and involves a mathematical vector calculation.

The main causal factor of myocardial CV is intercellular gap junction function. Atrial fibrosis and fibroblast can penetrate the extracellular matrix collagen content resulting in a slowing in the conduction of electrical properties, which is an important component of the arrhythmogenic substrate in patients with AF [23]. Regional CV can decline in the substrate of AF, and this discontinuous and electrical heterogeneity among the inter-cells may induce spontaneous abnormal electric activity [24]. Such fibrotic areas and sites of the LVA can correlate to each other [6, 25], and the significant slowing of local conduction in damaged tissue associated with fractionated or double potentials [21]. Consistently, our study demonstrated the mild correlation between LA-CV and LA-LVA, especially in the anterior wall rather than posterior LA wall. The anterior LA wall also showed a lower model LA-CV and larger LA-LVA compared with the posterior wall. Possible reasons for these findings are as follows. First, mechanical pressure exerted by an anatomically deviated or expanded ascending aorta course could be responsible for the development of the LVA on the anterior LA wall. The distribution of the LVA related to these anatomical features has been reported as possibly associated with AF appearance [26], and previous reports have shown that the mean voltage in the anterior LA wall was significantly lower than that in the posterior wall in patients with AF, which might be linked to prognosis after catheter ablation [27–29]. Second, conduction of the anterior propagation wave perpendicularly crosses the sept-pulmonary bundle and is attributed to a physiological slow anterior LA-CV [30]. The CV on the anterior wall may be more sensitive to the damage of atrial tissue and is likely to decrease with reference to the existence of the LVA.

Although both the anterior model LA-CV and anterior LA-LVA were associated with AF recurrence in the present study, the value for each parameter was not definitive individually, but it could be more effective when combined. Even though weakly, the LA-CV model was significantly correlated with the LA-LVA, and this intermix method might not always be persuasive because such a combined parameter is possible only to represent one parameter’s outcome. Indeed, previous studies reported that local atrial CV within the LVA was significantly slower than that in no LVA [21] and that both parameters could be just different manifestations of the same pathophysiological finding [31]. However, in the present study, patients with an anterior LA-LVA of ≤ 15.5 cm^2^ had a different distribution pattern between the two groups separated according to the cut-off point of the anterior model LA-CV (groups 1 and 2), while they have the same classification area of the voltage amplitude. Moreover, survival-free AF recurrence for one year was significantly different between the two groups. These results suggest that the size of LA-LVA is not an absolute factor associated with AF recurrence after ablation, and the LA-CV represented by the distinct distribution of the LVA, patchy and concentric patterns, could impact AF recurrence, especially in cases with less presence of LVA. This indicates that the “quality” of arrhythmogenic substrate factors is essential as well as the “quantity” of the less presence of LVA, while the difference in the LA-CV between groups 3 and 4 with large LVA may be the extent of the size of the mLVA which was a “quantity” of the LVA.

Furthermore, the decreased LA-CV was found to be an important factor to stratify prognosis significantly, even in patients with paroxysmal AF who were unlikely to have the LVA in the LA body. There is no doubt that the recurrence of patients with paroxysmal AF is mainly due to PV reconnection after ablation, but slowing LA-CV might be an early recognition feature to estimate the AF substrate before the development of a LVA of less than 0.5 mV or be a distinct process for substrate formation, which could be related to the clinical outcome as significant difference in this study. An effective therapeutic approach for such an area with decreased CV but not impaired voltage amplitude is unclear, and further studies are needed to address these issues.

## Study limitations

This study has several limitations. This is a retrospective study conducted at a single institution, and the number of patients enrolled was relatively small. To demonstrate predictive value of the LA-CV more strongly, a prospective validation set of patients is required. The study population included both patients with paroxysmal and persistent AF, which may be confounders. Ideally, the population would be limited to patients with persistent AF who are likely to have LA-LVA on the LA body. A part of patients with AF recurrence did not have second ablation treatment, and the recurrent mechanisms were not studied enough. Most patients with persistent AF need to undergo cardioversion after PVI, but the cardiac electrophysiological properties after cardioversion are still controversial. Since the model LA-CVs were measured manually, variability in the measurements could have occurred. Furthermore, applying this method in the clinical setting might be challenging due to the time and effort required. During the determination of the points for the calculation of the LA-CV, the investigator reviewed the activation sequence of the color-coded map to determine the direction, which could raise a possible bias in the point selection. Localized slow conductions in total LA areas can also be visualized with a novel vector-based map in CARTO® 3 V7. Further analyses evaluating the agreement between the CV and vector-based map in CARTO® 3 V7 module will also be required. Conduction time was calculated during sinus rhythm, not under coronary sinus proximal pacing, which could not eliminate the influence of the sinus rate or activation through the Bachman bundle. The LA-LVA distribution pattern was somewhat difficult to assess and subjective; thus, a strict definition is required in the future. Due to the limited performance of follow-up examinations (e.g., 12-lead electrocardiography, 24 h Holter monitoring) and rhythm assessment in our study, the prevalence of asymptomatic AF recurrence may have been underestimated [32].

## Conclusion

The low anterior model LA-CV was significantly associated with AF recurrence after catheter ablation, and combination with LA-LVA could improve the predictive value of outcomes. The decreased LA-CV was affected by the detailed amplitude and distribution pattern of the LVA, in addition to the total size of the LVA. The two electrical parameters are important for recognizing the cause of heterogeneity of the AF substrate. Further studies are required to validate the utility of the LA-CV concept and the application of the ablation strategy for AF.

## Electronic supplementary material

Below is the link to the electronic supplementary material.

Supplementary Material 1: Figure 1: Distribution patterns of the LVA in the left atrium. Figure 2: Distributions of the area classified by mean voltage amplitude of anterior LVA (A) and distribution patterns of the anterior LVA (B) among the four groups separated by the cut-off values of the CV and LVA.

## Data Availability

The datasets generated and/or analyzed during the current study are not publicly available due to ethical principles but are available from the corresponding author on reasonable request.

## Abbreviations

AF: atrial fibrillation
AFL: atrial flutter
AUC: areas under the curve
CI: confidence interval
CT: computed tomography
CV: conduction velocity
HR: hazard ratio
LA: left atrium
LAA: left atrial appendage
LVA: low voltage area
NPV: negative predictive value
PPV: positive predictive value
PV: pulmonary vein
PVI: pulmonary vein isolation
ROC: receiver operating characteristic
