# Supplementary material for: Regional left atrial conduction velocity in the anterior wall is associated with clinical recurrence of atrial fibrillation after catheter ablation: efficacy in combination with the ipsilateral low voltage area
Source: BMC Cardiovasc Disord. 2022 Nov 1;22:457. doi: 10.1186/s12872-022-02881-6 (PMC9628089; doi:10.1186/s12872-022-02881-6)
Supplement: Supplementary file 1 — Supplementary Material 1: Figure 1: Distribution patterns of the LVA in the left atrium. Figure 2: Distributions of the area classified by mean voltage amplitude of anterior LVA (A) and distribution patterns of the anterior LVA (B) among the four groups separated by the cut-off values of the CV and LVA. [file 12872_2022_2881_MOESM1_ESM.docx]

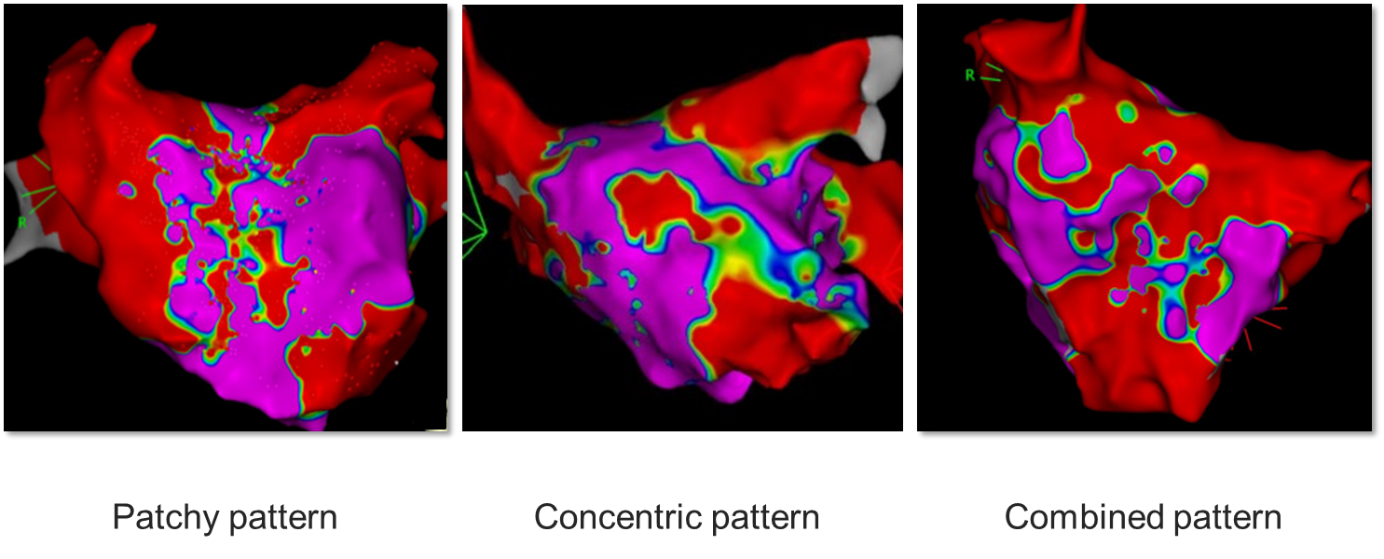


**Supplemental Figure 1**

Distribution patterns of the LVA in the left atrium. Three patterns were defined as patchy, concentric, and combined. LVA, low voltage area.


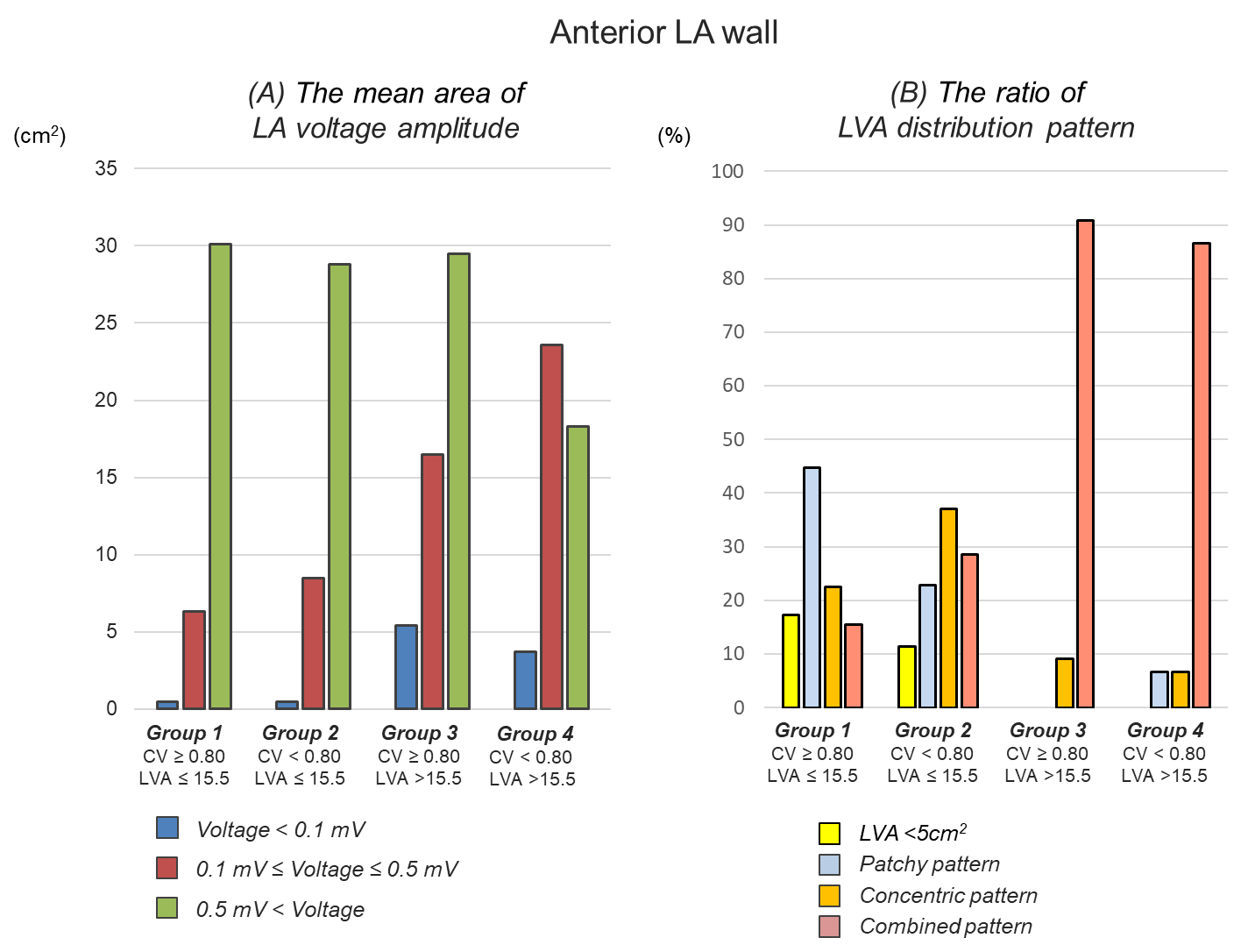


**Supplemental Figure 2**

Distributions of the area classified by mean voltage amplitude of anterior LVA (A) and distribution patterns of the anterior LVA (B) among the four groups separated by the cut-off values of the CV and LVA.

CV, conduction velocity; LVA, low voltage area.
